# Phase-segregated NiP_*x*_@FeP_*y*_O_*z*_ core@shell nanoparticles: ready-to-use nanocatalysts for electro- and photo-catalytic water oxidation through *in situ* activation by structural transformation and spontaneous ligand removal†
†Electronic supplementary information (ESI) available: Experimental details, additional characterization and results. See DOI: 10.1039/c8sc00420j


**DOI:** 10.1039/c8sc00420j

**Published:** 2018-04-30

**Authors:** Masaki Saruyama, Sunwon Kim, Toshio Nishino, Masanori Sakamoto, Mitsutaka Haruta, Hiroki Kurata, Seiji Akiyama, Taro Yamada, Kazunari Domen, Toshiharu Teranishi

**Affiliations:** a Institute for Chemical Research , Kyoto University , Gokasho , Uji , Kyoto 611-0011 , Japan . Email: saruyama@scl.kyoto-u.ac.jp ; Email: teranisi@scl.kyoto-u.ac.jp; b Department of Chemistry , Graduate School of Science , Kyoto University , Gokasho , Uji , Kyoto 611-0011 , Japan; c Mitsubishi Chemical Group Science and Technology Research Center, Inc. , 1000 Kamoshida-cho, Aoba-ku , Yokohama 227-8502 , Japan; d Japan Technological Research Association of Artificial Photosynthetic Chemical Process (ARPChem) , 7-3-1 Hongo, Bunkyo-ku , Tokyo 113-8656 , Japan; e Department of Chemical System Engineering , The University of Tokyo , 7-3-1 Hongo, Bunkyo-ku , Tokyo 113-8656 , Japan

## Abstract

The high overpotential of the oxygen evolution reaction is a critical issue to be overcome to realize efficient overall water splitting and enable hydrogen generation powered by sunlight. Homogeneous and stable nanoparticles (NPs) dispersed in solvents are useful as both electrocatalysts and cocatalysts of photocatalysts for the electro- and photo-catalytic oxygen evolution reaction, respectively, through their adsorption on various electrode substrates. Here, phase-segregated NiP_*x*_@FeP_*y*_O_*z*_ core@shell NPs are selectively synthesized by the reaction of Fe(CO)_5_ with amorphous NiP_*x*_ seed-NPs. The NiP_*x*_@FeP_*y*_O_*z*_ NPs on conductive substrates exhibit higher electrocatalytic activity in the oxygen evolution reaction than those of other metal phosphide-based catalysts. The NiP_*x*_@FeP_*y*_O_*z*_ NPs can also be used as a cocatalyst of an anodic BiVO_4_ photocatalyst to boost the photocatalytic water oxidation reaction. The excellent catalytic activity and high stability of the NiP_*x*_@FeP_*y*_O_*z*_ NPs without any post-treatments are derived from *in situ* activation through both the structural transformation of NiP_*x*_@FeP_*y*_O_*z*_ into mixed hydroxide species, (Ni, Fe)O_*x*_H_*y*_, and the spontaneous removal of the insulating organic ligands from NPs to form a smooth and robust (Ni, Fe)O_*x*_H_*y*_/substrate heterointerface during the oxygen evolution reaction.

## Abstract 2

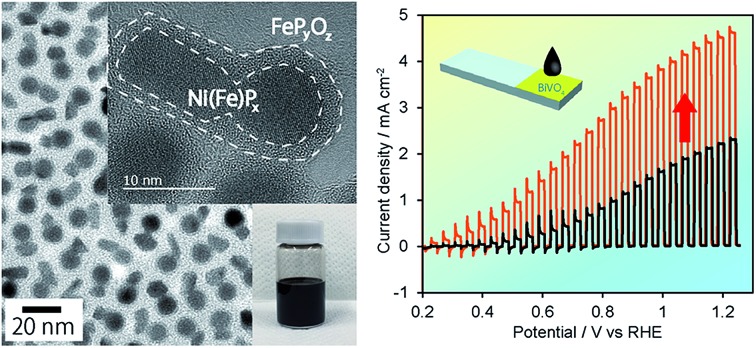
The phase-segregated NiP*_x_*@FeP*_y_*O*_z_* core@shell NPs act as a colloidally stable, ready-to-use, and excellent OER active transition metal phosphide-based catalyst.

## Introduction

Hydrogen evolution by efficient and sustainable electrolysis of water is desirable for mass-production of hydrogen as a clean energy source.^1^ The water splitting reaction consists of two half reactions: the oxygen evolution reaction (OER) and the hydrogen evolution reaction. The OER is considered to be the bottle-neck in the water splitting reaction because the OER typically requires a multistep four-electron process for O–O bond formation, which is kinetically slow. A high overpotential for the OER is required in water electrolysis, even with the use of rare and expensive metal catalysts, such as Ir and Ru.^2^ These factors limit the mass-production of water electrolysis devices. Currently, extensive explorations have been made into earth-abundant, low cost, and highly active materials, with a focus on non-noble transition-metal-based materials.^3^ In particular, transition metal phosphides have attracted much attention as highly efficient earth-abundant electrocatalysts for the OER.^4^

The most recently developed OER catalysts are micrometer-sized materials, in the form of powders,^5^ thin films,^6^ and microstructures grown on conductive substrates.^7^ Nano-sized OER catalysts have also been widely studied because they exhibit remarkable activities owing to their large specific surface areas.^8^ Well-dispersed OER catalyst nanoparticles (NPs) in solvents are considered to be particularly effective, because they can be used to modify various kinds of substrates, including conductive electrodes and photocatalyst semiconductors by simple deposition methods.^8^,^9^ Such catalyst NPs usually require a ligand removal process after deposition. Hence, OER catalyst NP “ink” systems, which do not require ligand removal processes, are attractive for developing both catalyst/electrode and cocatalyst/photocatalyst hybrid systems on a large scale.

In our investigations of such OER catalysts, we found that the phase-segregated NiP_*x*_@FeP_*y*_O_*z*_ core@shell NPs are colloidally stable and efficient OER active transition metal phosphide-based catalysts. The NiP_*x*_@FeP_*y*_O_*z*_ NPs could be adsorbed on various kinds of substrates by simple deposition methods. The resulting composites exhibited high and stable OER activity without the need for any post-treatments due to *in situ* activation of NiP_*x*_@FeP_*y*_O_*z*_ NPs. NiP_*x*_@FeP_*y*_O_*z*_ NP-loaded carbon substrates exhibited an OER overpotential of 0.25 V at 10 mA cm^–2^ in 0.1 M KOH. Adsorption of NiP_*x*_@FeP_*y*_O_*z*_ NPs also greatly enhanced the photocatalytic activity and durability of BiVO_4_, suggesting that the NiP_*x*_@FeP_*y*_O_*z*_ NPs can also be used to fabricate photocatalyst/cocatalyst hybrid systems on a large scale.

## Results and discussion

**Fig. 1:**
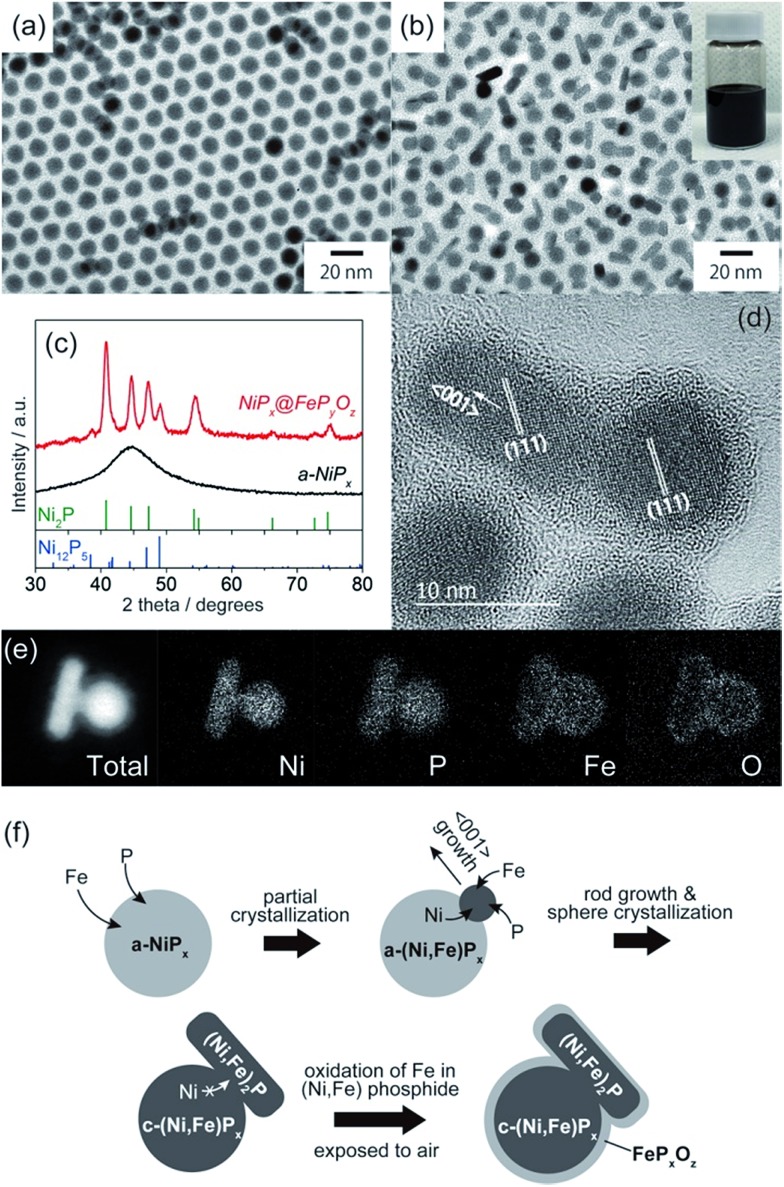
TEM images of (a) a-NiP_*x*_ NPs and (b) NiP_*x*_@FeP_*y*_O_*z*_ NPs. The inset shows a hexane dispersion of the NiP_*x*_@FeP_*y*_O_*z*_ NPs stored for more than 6 months. (c) XRD patterns of a-NiP_*x*_ NPs and NiP_*x*_@FeP_*y*_O_*z*_ NPs. (d) HRTEM image of NiP_*x*_@FeP_*y*_O_*z*_ NPs. (e) STEM-EDS mapping images of a NiP_*x*_@FeP_*y*_O_*z*_ NP. (f) Schematic of the formation mechanism of NiP_*x*_@FeP_*y*_O_*z*_ NPs.

NiP_*x*_@FeP_*y*_O_*z*_ core@shell NPs were synthesized through the reaction of a-NiP_*x*_ NPs and Fe(CO)_5_ (see ESI† for details). The a-NiP_*x*_ seed-NPs were 11.3 ± 0.7 nm in size (Fig. 1a) and their X-ray diffraction (XRD) pattern exhibited only one broad peak at 45°, indicating the amorphous structure of the NPs (Fig. 1c).^10^ After heating the a-NiP_*x*_ NPs with Fe(CO)_5_ in a mixture of 1-octadecene, oleylamine, and tri-*n*-octylphosphine (TOP) at 270 °C for 1 h, the spherical a-NiP_*x*_ NPs were transformed into a unique anisotropic structure, in which spherical particles (9.6 ± 0.7 nm) were connected with rod-shaped particles (10.8 ± 3.0 nm × 6.3 ± 1.2 nm) as shown in the transmission electron microscope (TEM) image (Fig. 1b). An XRD pattern of the resulting NPs featured diffraction peaks assigned to a mixture of the major Ni_2_P and minor Ni_12_P_5_ phases (Fig. 1c). Although no peaks from the Fe compounds were observed, we confirmed the presence of Fe [Ni/Fe = 78/22 (mol mol^–1^)] by X-ray fluorescence (XRF) analysis. High-resolution TEM (HRTEM) observations showed that both the spherical and rod-shaped phases were mainly composed of the Ni_2_P phase (Fig. 1d), and the Ni_12_P_5_ phase was rarely observed in our measurements (only one among eighteen NPs, Fig. S1†). These HRTEM images were consistent with the XRD results. The HRTEM images also show the presence of an amorphous shell layer surrounding the NiP_*x*_ core. Scanning TEM-energy dispersive X-ray spectroscopy (STEM-EDS) mapping of a single NP revealed that the elements Ni and P were mainly located at the core, and the elements Fe, O, and P were located at the shell, and therefore we describe the resulting NPs as NiP_*x*_@FeP_*y*_O_*z*_ NPs (Fig. 1e).

The structural evolution of the NiP_*x*_@FeP_*y*_O_*z*_ NPs was monitored during synthesis. At 10 min, large NiP_*x*_ NPs with small spherical domains were observed (Fig. S2a†). As the reaction proceeded, these small domains grew larger. The XRD patterns indicate that a-NiP_*x*_ started to change into crystalline NiP_*x*_ (c-NiP_*x*_) phases, including Ni_2_P and Ni_12_P_5_ at 30 min. The peak intensities increased until 60 min (Fig. S2b†). The Fe/Ni molar ratios of the NiP_*x*_@FeP_*y*_O_*z*_ NPs increased as the reaction progressed, indicating that Fe atoms were gradually incorporated into a-NiP_*x*_ seed-NPs (Fig. S3†).

The effects of Fe(CO)_5_ on the transformation of the a-NiP_*x*_ NPs were also studied. Without Fe(CO)_5_, the a-NiP_*x*_ NPs crystallized in a spherical shape (Fig. S4†), indicating that the Fe atoms induced a partial transformation of the spherical a-NiP_*x*_ into a rod-shaped phase.

In an XRD pattern of the NiP_*x*_@FeP_*y*_O_*z*_ NPs, the (111) peak slightly shifted from the position of the pure Ni_2_P phase owing to Fe incorporation into Ni_2_P.^11^ From the (111) peak position of NiP_*x*_@FeP_*y*_O_*z*_ NPs at 40.97°, the Fe content in the core of NiP_*x*_@FeP_*y*_O_*z*_ NPs was estimated to be ∼5 mol% (Fig. S5†).^11^ XRF analysis revealed the Ni : Fe molar ratio of the c-NiP_*x*_ cores to be 96 : 4, through selective etching of the FeP_*y*_O_*z*_ shells by H_2_SO_4_. These results agreed with the XRD results (Fig. S6†). As previously reported, Ni_2–*x*_Fe_*x*_P NPs tend to form rods or wires, because the Ni_2–*x*_Fe_*x*_P phase preferentially grows along the P phase preferentially grows along the 〈001〉 direction.001P phase preferentially grows along the 〈001〉 direction. direction.^12^ HRTEM images of the NiP_*x*_@FeP_*y*_O_*z*_ NPs showed that the long axis of the rod domains also grew in the direction of the NPs showed that the long axis of the rod domains also grew in the direction of the 〈001〉 plane for Ni001 NPs showed that the long axis of the rod domains also grew in the direction of the 〈001〉 plane for Ni plane for Ni_2_P (Fig. 1d); thus, both incorporation of Fe into NiP_*x*_ and the crystallization contributed to the anisotropic growth of the NiP_*x*_ NPs.

From these results, we propose the following mechanism for the formation of NiP_*x*_@FeP_*y*_O_*z*_ NPs (Fig. 1f). Initially, Fe atoms become incorporated into the a-NiP_*x*_ NPs. When the a-NiP_*x*_ NPs partially crystallize into small Ni_2–*x*_Fe_*x*_P domains, they grow along the P domains, they grow along the 〈001〉 direction to form rod structures. During rod growth, Ni atoms are supplied from spherical a-NiP001P domains, they grow along the 〈001〉 direction to form rod structures. During rod growth, Ni atoms are supplied from spherical a-NiP direction to form rod structures. During rod growth, Ni atoms are supplied from spherical a-NiP_*x*_ phases. When the a-NiP_*x*_ phases are completely crystallized, the Ni migration and structural transformations terminate. As a result, anisotropic spherical and rod-shaped NPs are formed. Finally, the FeP_*y*_O_*z*_ shells are generated by surface oxidation during the purification step in air.

**Fig. 2:**
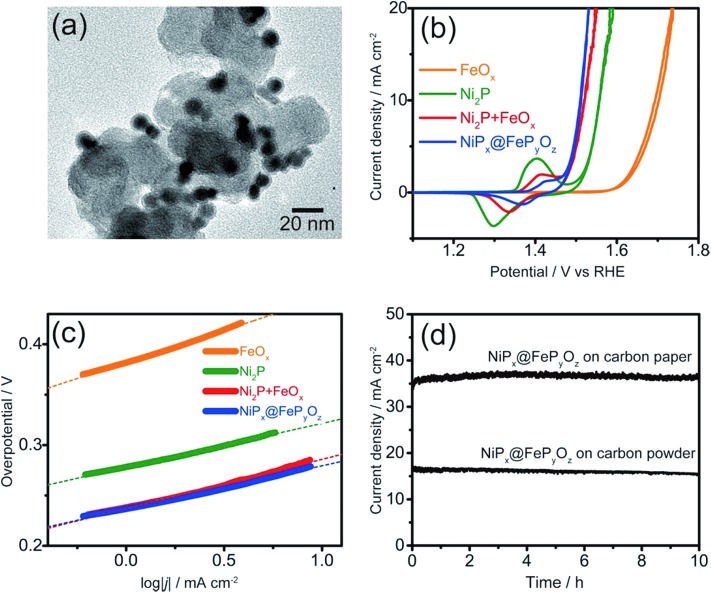
(a) TEM image of NiP_*x*_@FeP_*y*_O_*z*_ NP-loaded carbon powder. (b) Cyclic voltammograms and (c) Tafel plots of NiP_*x*_@FeP_*y*_O_*z*_, FeO_*x*_, Ni_2_P, and FeO_*x*_ + Ni_2_P NPs supported on carbon powder in 0.1 M KOH at 10 mV s^–1^. (d) CA curves of the NiP_*x*_@FeP_*y*_O_*z*_ NPs (0.075 mg cm^–2^) loaded on carbon paper and carbon powder at an overpotential of 0.35 V and 0.30 V in 0.1 M KOH, respectively.

The NiP_*x*_@FeP_*y*_O_*z*_ NPs were stable in a hexane dispersion for more than half a year, and could be easily adsorbed on various kinds of substrates, including carbon powder, carbon paper, and FTO coated glass, by simple mixing or deposition methods (see ESI† for details). Their OER catalytic activities were examined without any post-treatments such as annealing or ligand exchange to remove the organic ligands. Cyclic voltammetry (CV) curves of the NiP_*x*_@FeP_*y*_O_*z*_ NPs, Ni_2_P NPs, FeO_*x*_ NPs, and a mixture of Ni_2_P NPs and FeO_*x*_ NPs [Ni_2_P + FeO_*x*_, Ni/Fe = 77/23 (mol mol^–1^)] loaded carbon powder in 0.1 M KOH (Fig. 2a, S7 and S8†) are shown in Fig. 2b. Interestingly, the simply mixed Ni_2_P + FeO_*x*_ NPs showed a lower overpotential than those of Ni_2_P and FeO_*x*_ NPs; however, the NiP_*x*_@FeP_*y*_O_*z*_ NPs exhibited a further lower overpotential of 0.28 V at 10 mA cm^–2^ (0.36 V without *iR* compensation, Fig. S9†). NiP_*x*_@FeP_*y*_O_*z*_ NPs with different Ni/Fe molar ratios (85/15 and 72/28) were synthesized by changing the reaction time and showed overpotentials of 0.29 and 0.30 V at 10 mA cm^–2^ (Fig. S10†). Thus, the NiP_*x*_@FeP_*y*_O_*z*_ NPs with a Ni/Fe molar ratio of 78/22 were found to be the best OER catalyst in this work. The overpotential of 0.28 V is lower than those of many other previously reported metal phosphide-based OER catalysts (Table S1†).8a,^13^ More importantly, the amount of loaded NPs (0.02 mg cm^–2^) was much smaller than those of other catalysts, further confirming the excellent OER activity of the NiP_*x*_@FeP_*y*_O_*z*_ NPs.8a,^13^ The Tafel slope of the NiP_*x*_@FeP_*y*_O_*z*_ NPs, 43 mV dec^–1^, was smaller than those of Ni_2_P (44 mV dec^–1^), FeO_*x*_ (64 mV dec^–1^), and Ni_2_P + FeO_*x*_ (48 mV dec^–1^) NPs (Fig. 2c), and was also better than those of most of previously reported phosphide-based OER catalysts.8a,^13^ These results suggest favorable OER kinetics for the NiP_*x*_@FeP_*y*_O_*z*_ NPs. We also checked the NP loading amount dependent OER activity of NiP_*x*_@FeP_*y*_O_*z*_ NPs/carbon paper composites (Fig. S11†). The densely loaded electrode (0.5 mg cm^–2^) showed an overpotential of 0.25 V at 10 mA cm^–2^, which is better than those of other transition metal phosphide OER catalysts reported recently (Table S1†). Additionally, long-term chronoamperometry (CA) testing of NiP_*x*_@FeP_*y*_O_*z*_ NP-loaded carbon powder and paper showed no major decrease of the current densities during the continuous OER for 10 h, indicating the high OER operational stability of the NiP_*x*_@FeP_*y*_O_*z*_ NPs (Fig. 2d and S11c†).

To understand the origin of the high OER activity of the NiP_*x*_@FeP_*y*_O_*z*_ NPs, we performed XRF and X-ray photoelectron spectroscopy (XPS) on the NiP_*x*_@FeP_*y*_O_*z*_ NP-loaded carbon paper before and after the OER (100 cycles of CV in 0.1 M KOH). The XRF results of the NiP_*x*_@FeP_*y*_O_*z*_ NPs after the OER revealed a considerable decrease of the element P, while the Ni : Fe molar ratio was maintained. Thus, P was selectively eliminated during the OER (Fig. S12†). Core level XPS spectra of the NiP_*x*_@FeP_*y*_O_*z*_ NPs before and after the OER are shown in Fig. S13.† Before the OER, the Ni 2p peak intensity was small because of coverage of the NiP_*x*_ cores by FeP_*y*_O_*z*_ shells (Fig. S13a†). After the OER, the Ni 2p peak at 857 eV clearly emerged, which was attributed to the Ni 2p_3/2_ peak of Ni oxide or hydroxide.^14^ This result also indicates that Ni^2+^ ions were exposed to the surface of catalysts during the OER. For the case of P, before the OER, P 2p peaks appeared at 133 and 130 eV corresponding to PO_4_^3–^ species in the FeP_*y*_O_*z*_ shells and P^0^ in the partially exposed NiP_*x*_ cores, respectively (Fig. S13b†).^15^ After the OER, these P 2p peaks completely disappeared owing to the elimination of P, which is consistent with the XRF results. The Fe 2p peak at 711 eV before the OER could be assigned to Fe oxide or phosphate in FeP_*y*_O_*z*_ shells. This peak markedly shifted to 714 eV, corresponding to Fe (oxy)hydroxide, after the OER (Fig. S13c†).^16^ In the case of O, before the OER, the O 1s peak at 531 eV, attributed to metal oxide or phosphate, shifted to 532 eV, which could be assigned to metal (oxy)hydroxide after the OER (Fig. S13d†).^17^ We conclude from the XPS results that the NiP_*x*_@FeP_*y*_O_*z*_ NPs were transformed into (Ni, Fe)O_*x*_H_*y*_ during the OER. After the transformation, the elements Ni and Fe were homogeneously distributed over the entire catalyst surface, and P was dissolved. XPS spectra measured after Ar bombardment also indicated this transformation occurred (see Fig. S13† for details). Fe-doped NiO_*x*_H_*y*_ has been reported to have much higher activity than pure NiO_*x*_H_*y*_, because the Fe ions surrounded by the Ni ions behave as active centres for the OER.^18^ Because the active Ni species in the NiP_*x*_@FeP_*y*_O_*z*_ NPs are covered with the FeP_*y*_O_*z*_ shell, the catalytic activity for the OER should be low, as shown in the case of the FeO_*x*_ NPs. However, the elimination of element P and the subsequent structural transformation of NiP_*x*_@FeP_*y*_O_*z*_ into (Ni, Fe)O_*x*_H_*y*_ create the OER active sites.

The formation of (Ni, Fe)O_*x*_H_*y*_ was further supported by the CV results. The CV of NiP_*x*_@FeP_*y*_O_*z*_ NPs showed a smaller redox peak area at 1.48 V *vs.* RHE than that of Ni_2_P (Fig. 2b). This result implies that Fe diffused into the NiO_*x*_H_*y*_, because the Fe cations doped into NiO_*x*_H_*y*_ suppressed oxidation of Ni^2+^.^19^ Although, Ni_2_P + FeO_*x*_ NPs also showed a smaller redox peak area of Ni^2+^ than that of Ni_2_P, and the peak was larger than that of the NiP_*x*_@FeP_*y*_O_*z*_ NPs. This suggests that the Fe diffusion was incomplete for the case of Ni_2_P + FeO_*x*_ NPs because the Ni_2_P and FeO_*x*_ NPs were spatially separated. Thus, the direct contact of Ni- and Fe-containing phases in NiP_*x*_@FeP_*y*_O_*z*_ NPs was advantageous for fabricating homogeneously mixed metal compound catalysts.

**Fig. 3:**
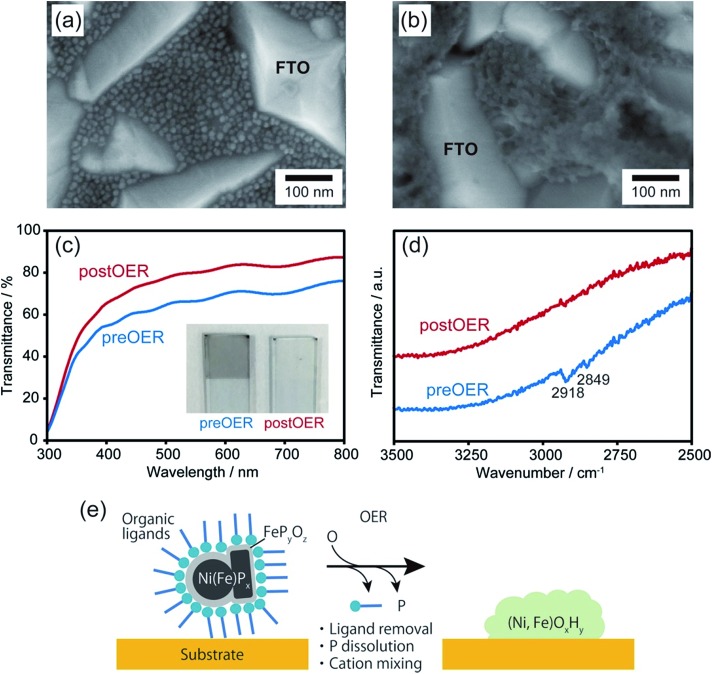
SEM images of NiP_*x*_@FeP_*y*_O_*z*_ NP coated FTO glass (a) before and (b) after 30 CV cycles in 0.1 M KOH. (c) Transmittance and (d) FT-IR spectra of NiP_*x*_@FeP_*y*_O_*z*_ NP coated FTO glass (blue) before and (red) after 30 CV cycles in 0.1 M KOH. The inset in (c) shows the photograph of NiP_*x*_@FeP_*y*_O_*z*_ NP coated FTO glass. (e) Schematic illustration of the transformation of NiP_*x*_@FeP_*y*_O_*z*_ NPs into (Ni, Fe)O_*x*_H_*y*_ during the OER.

Upon chemical transformation, the morphology of the NiP_*x*_@FeP_*y*_O_*z*_ NPs on the substrates changed to a film-like structure owing to fusion of the NiP_*x*_@FeP_*y*_O_*z*_ NPs (Fig. 3a, b and S14†), which led to the drastic change of their absorption spectrum. The transmittance of the NiP_*x*_@FeP_*y*_O_*z*_ NPs film on the FTO-coated glass became higher after 30 CV cycles in 0.1 M KOH owing to the formation of hydroxide species with a low absorption coefficient (Fig. 3c and S15†).^20^ Highly transparent catalysts in the visible region are particularly beneficial as cocatalysts for photocatalysts, because they do not obstruct incident light from reaching the photocatalysts. Furthermore, Fourier transform infrared (FT-IR) spectroscopy of the NiP_*x*_@FeP_*y*_O_*z*_ NPs on FTO before and after CV revealed that the C–H stretching vibration peaks at 2849 and 2918 cm^–1^ disappeared after the CV scans, indicating that the organic ligands (oleylamine and TOP) were completely removed during CV (Fig. 3d). This spontaneous removal of insulating ligands is a major advantage of the NiP_*x*_@FeP_*y*_O_*z*_ NPs as both a ready-to-use electrocatalyst and as a cocatalyst for photocatalysts, because post-treatment processes can be omitted to form NP/substrate heterointerfaces directly (Fig. 3e).

**Fig. 4:**
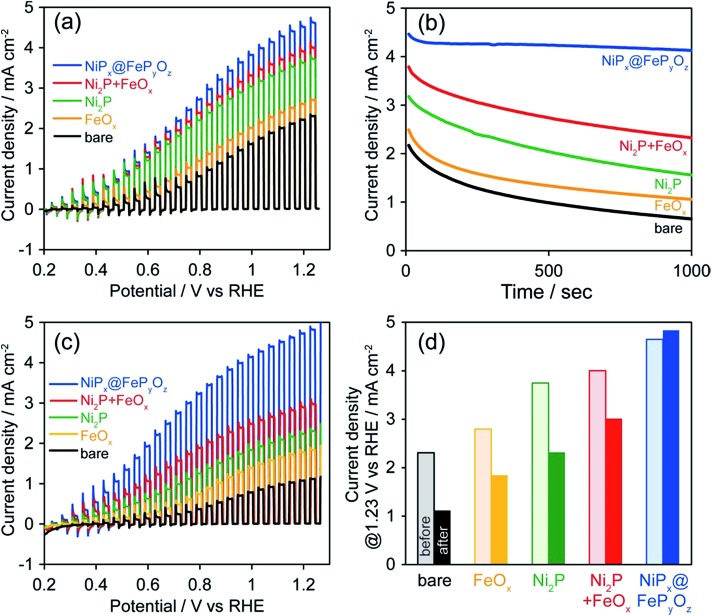
(a) Photocurrent density curves before CA, (b) CA curves, and (c) photocurrent density curves after CA, and (d) photocurrent densities at 1.23 V *vs.* RHE before and after CA of bare and NP-loaded BiVO_4_ in 0.125 M K_2_B_4_O_7_ at 1.23 V *vs.* RHE (300 W Xe lamp with a <385 nm cut filter).

To prove the versatile application of NiP_*x*_@FeP_*y*_O_*z*_ NPs, we applied the NiP_*x*_@FeP_*y*_O_*z*_ NPs as an OER cocatalyst with an anodic semiconductor photocatalyst to boost photocatalytic water oxidation (light source: 300 W Xe lamp with a 385 nm short-cut filter). A hexane solution of FeO_*x*_, Ni_2_P, Ni_2_P + FeO_*x*_, or NiP_*x*_@FeP_*y*_O_*z*_ NPs was deposited on porous BiVO_4_ film electrodes^21^ and spin-dried, followed by washing with ethanol (Fig. S16†). Note that no post-treatment processes were performed in the following measurements. Linear sweep voltammetry (LSV) measurements with chopped light in 0.125 M K_2_B_4_O_7_, in Fig. 4a, showed that the loading of the NPs enhanced the photocurrents and that the NiP_*x*_@FeP_*y*_O_*z*_ NP-loaded BiVO_4_ film electrode exhibited the largest photocurrent among the NPs used in this work. CA measurements with continuous light irradiation (@1.23 V *vs.* RHE) showed that the NiP_*x*_@FeP_*y*_O_*z*_ NPs loaded on BiVO_4_ possessed the highest durability to continuous water photo-oxidation and maintained 92% of their photocurrent after 1000 s (Fig. 4b and S17†). However, the current densities of the bare, Ni_2_P, FeO_*x*_, and Ni_2_P + FeO_*x*_ NP-loaded BiVO_4_ decreased to 30, 49, 43, and 61% of their initial current densities, respectively. Interestingly, we found that the photocurrent of the NiP_*x*_@FeP_*y*_O_*z*_ NP-loaded BiVO_4_ was further enhanced after the CA measurement (Fig. 3c, d and S18e†), because the CA measurement promoted the transformation of the NiP_*x*_@FeP_*y*_O_*z*_ NPs into (Ni, Fe)O_*x*_H_*y*_. We also confirmed the transformation of NiP_*x*_@FeP_*y*_O_*z*_ NPs and enhanced OER activity in 0.125 M K_2_B_4_O_7_ (Fig. S19†). Conversely, the photocurrents of bare and other NP-loaded BiVO_4_ electrodes decreased after CA measurements (Fig. 3c, d and S18a–d†). This effect was likely caused by degradation of BiVO_4_ owing to accumulation of photogenerated holes in BiVO_4_.^22^ Namely, slow water oxidation kinetics at the surface of BiVO_4_ led to photo-corrosion under continuous light irradiation. By loading efficient OER cocatalysts onto the photoanode, holes were immediately consumed to oxidize water, preventing photo-corrosion and improving stability. These results also indicate the excellent catalytic OER activity of NiP_*x*_@FeP_*y*_O_*z*_ NPs as a cocatalyst.

For practical use, the photoelectrochemical measurements of NiP_*x*_@FeP_*y*_O_*z*_ NPs/BiVO_4_ were also conducted under simulated sunlight (Fig. S20†). By loading NPs, the photocurrent density of NiP_*x*_@FeP_*y*_O_*z*_ NPs/BiVO_4_ at 1.23 V *vs.* RHE reached 2.3 mA cm^–2^, which is more than double that of bare BiVO_4_ (1.1 mA cm^–2^). Loading NiP_*x*_@FeP_*y*_O_*z*_ NPs increased the surface charge transfer efficiency (*η*_surface_) from 40% to 73% at 1.23 V *vs.* RHE (Fig. S20b†). Especially, the *η*_surface_ at lower potential, 0.6 V *vs.* RHE, was greatly improved from 9% to 58%, also proving the fast OER kinetics of NiP_*x*_@FeP_*y*_O_*z*_ NP cocatalyst. A long-term stability test was also conducted for each electrode, and the highest durability was 62% photocurrent retention in 3 h at 1.23 V *vs.* RHE (Fig. S20d†).

Generally, a robust cocatalyst layer on BiVO_4_ drastically improves both activity and durability (Table S2†).^21^ On the other hand, partial coverage of BiVO_4_ with nanosized particles or molecules tends to show limited improvement of BiVO_4_ stability (Table S2†).^22^ Because, in our case, the NiP_*x*_@FeP_*y*_O_*z*_ NPs partially attach to BiVO_4_, it seems to be insufficient to fully boost the activity of BiVO_4_. However, *η*_surface_ = 58% at 0.6 V *vs.* RHE is relatively high and the best durability (62% in 3 h) is better than that of partially covered BiVO_4_, despite the use of an ultimately simple and fast method (completed within ∼10 s under ambient conditions). However, there is plenty of room for further improvement of the photocatalyst performance. In addition to the OER kinetics on the surface of the cocatalyst, the hole transfer from the photocatalyst to the cocatalyst should be considered. Tuning the band structure of NiFe(OH)_*x*_ must be effective and may be realized by incorporating a foreign metal into NiP_*x*_@FeP_*y*_O_*z*_ NPs.^23^

Recent studies on efficient Ni–Fe oxide, hydroxide, or oxyhydroxide based OER electrocatalysts showed significantly small overpotentials less than 0.3 V at 10 mA cm^–2^.^24^ Most of these catalysts are in bulk form, such as micrometer scale NiFe layered double hydroxides,24a,b composites with carbon,24*c* and Ni foam.24*d* Such bulk electrocatalysts, however, are difficult to directly hybridize with semiconductor photocatalysts, because of the small interfacial contact area and the lack of a robust bond between electrocatalysts and photocatalysts by simple mixing. This would be the reason why excellent electrocatalyst/photocatalyst hybrid system combinations have been rarely reported. Our NiP_*x*_@FeP_*y*_O_*z*_ NPs allow us to readily form a number of durable NPs/substrate heterointerfaces through an *in situ* activation and provide excellent OER activity with various kinds of conductive and semiconductive substrates. This feature is a considerable advantage of our catalytic NPs in the fabrication of large scale electro- and photo-catalyst systems.

## Conclusions

In conclusion, we developed a selective synthesis of monodisperse, colloidally stable, and phase-segregated NiP_*x*_@FeP_*y*_O_*z*_ core@shell NPs with high OER activity. Using our NiP_*x*_@FeP_*y*_O_*z*_ NP ink, we loaded the NPs onto various conductive and semiconductor substrates and found excellent OER activity. We discovered that migration of Ni and Fe occurred between the phase separated NiP_*x*_ and FeP_*y*_O_*z*_ phases, which served as efficient OER active sites for the OER. This process also induced spontaneous removal of ligands and *in situ* formation of the NP/substrate heterointerfaces, which provided ready-to-use OER hybrid catalysts without the need for any post-treatments. We demonstrated that even phase-segregated structures could be transformed into homogeneous active phases, suggesting a new way to design efficient nanostructured catalysts.

## Conflicts of interest

There are no conflicts to declare.

## Supplementary Material

Supplementary information
