# Supplementary material for: Phase-segregated NiPx@FePyOz core@shell nanoparticles: ready-to-use nanocatalysts for electro- and photo-catalytic water oxidation through in situ activation by structural transformation and spontaneous ligand removal
Source: Chem Sci. 2018 Apr 30;9(21):4830–6. doi: 10.1039/c8sc00420j (PMC5982198; doi:10.1039/c8sc00420j)
Supplement: Supplementary file 1 [file SC-009-C8SC00420J-s001.pdf]

Electronic Supplementary Material (ESI) for Chemical Science.
This journal is © The Royal Society of Chemistry 2018

Electric Supplementary Information (ESI) for

Phase-Segregated NiP_x@FeP_yO_z Core@Shell Nanoparticles: Ready-to-Use Nanocatalysts for Electro- and Photo-Catalytic Water Oxidation through in-situ Activation by Structural Transformation and Spontaneous Ligand Removal

Masaki Saruyama,[a] Sunwon Kim,[b] Toshio Nishino,[a] Masanori Sakamoto,[a] Mitsutaka Haruta,[a] Hiroki Kurata,[a] Seiji Akiyama,[c,d] Taro Yamada,[e] Kazunari Domen,[e] and Toshiharu Teranishi*[a]*

[a] *Institute for Chemical Research, Kyoto University, Gokasho, Uji, Kyoto 611-0011, Japan. E-mail: saruyama@scl.kyoto-u.ac.jp, teranisi@scl.kyoto-u.ac.jp*

[b] *Department of Chemistry, Graduate School of Science, Kyoto University, Gokasho, Uji, Kyoto 611-0011, Japan*

[c] *Mitsubishi Chemical Group Science and Technology Research Center, Inc., 1000 Kamoshida-cho, Aoba-ku, Yokohama 227-8502, Japan*

[d] *Japan Technogical Research Association of Artificial Photosynthetic Chemical Process (ARPChem), 7-3-1 Hongo, Bunkyo-ku, Tokyo 113-8656, Japan*

[e] *Department of Chemical System Engineering, The University of Tokyo, 7-3-1 Hongo, Bunkyo-ku, Tokyo 113-8656, Japan*

Experimental Procedures

Chemicals

$Ni(acac)_2$ (95%), 1-octadecene (90%), Nafion perfluorinated resin solution (5 wt%), $VO(acac)_2$ (98%), KI (99+%), and p-benzoquinone (>98%) were purchased from Sigma-Aldrich Co., LLC. Oleylamine (C18 content 80–90%) was purchased from Acros Organics. $Fe(CO)_5$ (>95%) and tri-n-octylphosphine (TOP, >95%) were purchased from Kanto Chemical Co., Inc. $Bi(NO_3)_3\cdot 5H_2O$ (99.9%) was purchased from Wako Pure Chemical Industries, Ltd. Di-n-octyl ether (>95%) was purchased from Tokyo Chemical Industry Co., Ltd. All reagents were used without further purification.

Characterization

Transmission electron microscope (TEM) observations were performed on a JEM1011 (JEOL) operated at 100 kV. High-resolution TEM (HRTEM) and scanning TEM-energy dispersive X-ray spectroscopy (STEM-EDS) observations were performed on a JEM-ARM200F operated at 200 kV. Scanning electron microscope (SEM) observations were performed on a S-4800 (Hitachi). X-ray diffraction (XRD) patterns were measured on PANalytical X'Pert Pro MPD with CuKα radiation (λ = 1.542 Å) operated at 45 kV and 40 mA. X-ray photoelectron spectroscopy (XPS) measurements were conducted in an ultrahigh vacuum combined system equipped with a hemispherical electron analyzer and a Mg Kα X-ray source (1253.6 eV). The detail has been described in a previous paper [1]. Transmittance measurements were acquired on a U-3310 spectrophotometer (Hitachi). X-ray fluorescence (XRF) elemental analysis was performed on an Element Analyzer JSX-3202C (JEOL). Fourier Transform infrared (FT-IR) analyses were performed on a IR Prestige 21 (Shimadzu).

Synthesis of a-NiP$_x$ NPs

$Ni(acac)_2$ (1 mmol), 1-octadecene (4.5 mL), and oleylamine (6.4 mL) were mixed and heated to 80 °C under a N_2 atmosphere to form a blue-green transparent solution. TOP (2 mL) was injected and the mixture was heated to 230 °C at 10 °C min^{-1}, and maintained at that temperature

for 30 min. The reaction solution was then cooled to room temperature and purified with ethanol. The precipitated solid NPs were redispersed in hexane and stored for further use.

Synthesis of NiP_x@FeP_yO_z NPs

A 34-mg portion of a-NiP_x NPs was dispersed in a mixture of 1-octadecene (9 mL) and oleylamine (1.9 mL). After repeated degassing-refilling with N_2, TOP (2 mL) solution containing $Fe(CO)_5$ (0.4 mmol) was injected into the mixture at room temperature. The mixed solution was heated to 270 °C for 60 min. After the reaction, the solution was cooled to room temperature and purified with ethanol. The precipitated solid NPs were redispersed in hexane and stored for further use.

Synthesis of Ni_2P NPs

a-NiP_x NPs (85 mg) was dissolved in a mixture of di-*n*-octyl ether (9 mL) and oleylamine (1.9 mL). The mixture was heated to 270 °C at 10 °C min^{-1} under a N_2 atmosphere. The solution was reacted at 270 °C for 60 min, cooled to room temperature, and purified with ethanol. The precipitated solid NPs were redispersed in hexane and stored for further use.

Synthesis of FeO_x NPs

The 5-nm FeO_x NPs were synthesized following a literature procedure [2]. Briefly, a mixture of $Fe(CO)_5$ and oleylamine (1:1 mol/mol) was heated to 80 °C at 2 °C min^{-1} and held at that temperature for 30 min under a N_2 atmosphere. A deep red solution of $Fe(CO)_5$-oleylamine complex was injected into 1-octadecene at 180 °C in a N_2 atmosphere. The solution was reacted at 180 °C for 30 min, cooled to room temperature, and then purified with ethanol. The precipitated solid NPs were redispersed in hexane and stored for further use.

Preparation of NP-loaded carbon powder catalysts

The electrocatalyst powder was prepared by mixing NPs and conductive carbon powder (XC-72, Cabot). Typically, XC-72 (8 mg) was dispersed in hexane (8 mL) by sonication, followed by the dropwise addition of hexane (2 mL) containing NPs (2 mg). Further sonication was

applied for 30 min. The catalyst powder was collected by centrifugation, washed with acetone twice, and dried under vacuum.

Preparation of carbon powder catalyst working electrodes

Catalyst powder (1 mg) was mixed with water (396 μL), 2-propanol (94 μL), and Nafion resin solution (10 μL), then sonicated for 30 min to form a homogenous catalyst slurry. The catalyst slurry was deposited on a glassy carbon rotating disk electrode 5 mm in diameter and dried by rotating at 700 rpm under ambient conditions.

Preparation of NP-loaded carbon paper electrodes

A 1 × 3 cm^2 piece of carbon paper (Toray, TGP-H-120) was washed with ethanol and acetone, and dried under vacuum. A hexane solution of NiP_x@FeP_yO_z NPs was dropped on the carbon paper (0.01, 0.075 or 0.5 mg cm^{-2}), washed with ethanol, and dried under vacuum.

Immobilization of NPs on FTO-coated glass electrodes

A 1.5 × 5 cm^2 piece of FTO-coated glass (AGC Fabritech) was washed with ethanol and acetone. The hexane solution of NiP_x@FeP_yO_z NPs (3 mg mL^{-1}, 50 μL) was dropped onto the FTO coated glass and spun at 1000 rpm for 10 s (the coated area was 2.25 cm^2).

Acid etching of NiP_x@FeP_yO_z NPs

The NiP_x@FeP_yO_z NPs-loaded carbon powder was mixed with 0.5 M H_2SO_4 aqueous solution (2 mL) and sonicated for 1 h. Then the powder was collected by centrifugation and washed with water and acetone. Finally, the powder was dried under vacuum for TEM and XRF measurements.

Preparation of $BiVO_4$ electrodes

We prepared porous $BiVO_4$ electrodes according to the method reported by Choi *et al.* [3]. Briefly, a BiOI film was deposited on an FTO coated glass substrate by immersing the substrate in a water/ethanol solution of $Bi(NO_3)_2$, KI, and p-benzoquinone, followed by the application of –0.1 V vs Ag/AgCl (3 M KCl) for 2 min (the deposited area was 1~1.5 cm^2). A DMSO solution of $VO(acac)_2$ was placed on the BiOI film, followed by annealing at 450 °C for 2 h in

air. The annealed electrode was immersed in 1 M NaOH for 30 min to remove V_2O_5 by-products, washed with deionized water, and dried under ambient conditions.

NP deposition on $BiVO_4$ electrodes

Before loading the NPs on the $BiVO_4$ photoelectrodes, photoelectrochemical (PEC) measurements of all $BiVO_4$ photoelectrodes were performed in 0.125 M $K_2B_4O_7$ (pH 9.4) with a 300 W Xe lamp with a >385 nm short-wavelength cut-off filter (see below), and photoelectrodes showing the similar photocurrents were selected for NP loading for comparison with the photocurrents precisely. The FTO surface of the $BiVO_4$ electrode was masked with scotch tape. A hexane solution of NPs (0.05, 0.25, 1 or 5 mg mL^{-1}, 25 μL) was dropped on the $BiVO_4$ film, and spun at 1000 rpm for 10 s. The NP-deposited electrodes were washed with ethanol and dried under ambient conditions.

Electrochemical measurements

The electrochemical measurements were performed in a three-electrode electrochemical cell with a ALS620C electrochemical analyzer (BAS) at room temperature (~25 °C). Hg/HgO (1 M NaOH) or Ag/AgCl (3 M NaCl) was used as a reference electrode in 0.1 M KOH or 0.125 M $K_2B_4O_7$ electrolyte, respectively. The reference electrodes were calibrated with an unused saturated calomel electrode in saturated NaCl aqueous solution. A Pt coil was used as a counter electrode. Prior to each measurement, the electrolyte was deaerated by bubbling with Ar for 20 min to remove oxygen. Cyclic voltammograms and chronoamperometry were conducted under a continuous Ar flow. In the case of a rotating disk electrode, the working electrode was rotated at 1600 rpm during measurements with a RRDE-3A (BAS). In the case of carbon paper and FTO coated glass electrodes, the working electrode was immersed and the electrolyte solution was stirred during the measurements. All displayed voltammograms except for Figure S9 and S11b were *iR*-collected to account for any uncompensated resistance. The uncompensated resistance was measured with a function of the analyzer based on a previously reported

technique [4]. The conversion between potentials vs. reference electrode (E_{ref}) and vs. reversible hydrogen electrode (RHE) (E_{RHE}) was performed with the equation below:

$$E_{RHE} = E\ (\text{vs. } E_{ref}) + E_{ref} + 0.059 \times \text{pH} - iR$$

PEC measurements

The PEC performance of the photoanodes was evaluated in a three-electrode configuration with a ALS620C electrochemical analyzer (BAS) and a light source (300 W Xe lamp, MAX-303, Asahi Spectra). Illumination was obtained by passing light from the source through a >385 nm short wavelength cut-off filter, and irradiated through the FTO side (back-side illumination). The power density of the incident light was ~190 mW cm^{-2} at the $BiVO_4$/FTO electrode measured by a thermal sensor (PS10, Coherent). The simulated sunlight was obtained using 300 W Xe lamp equipped with AM filter. Power density was adjusted to 100 mW cm^{-2} using standard Si solar cell (BS-500BK, BUNKOUKEIKI). Photocurrent measurements were performed in 0.125 M $K_2B_4O_7$ buffer solution (pH 9.4). Prior to each measurement, the electrolyte was deaerated by bubbling Ar for 20 min. Photocurrents were obtained with illumination and sweeping the potential in the positive direction at a scan rate of 10 mV s^{-1} or applying a constant bias. All measurements were performed with a Ag/AgCl (3 M KCl) reference electrode, and all results in this work are reported against RHE. The Ag/AgCl reference electrode was calibrated with an unused saturated calomel electrode in saturated NaCl solution. The conversion between potentials vs. Ag/AgCl and vs. RHE was performed using the equation below:

$$E_{RHE} = E\ (\text{vs. Ag/AgCl}) + E_{Ag/AgCl} + 0.059 \times \text{pH}$$

The surface charge transfer efficiency ($\eta_{surface}$) using the photocurrent of water (J_{water}) and sulfite ($J_{sulfite}$) oxidation according to the equation below.

$$\eta_{surface}\ \% = J_{water} / J_{sulfite} * 100\%$$

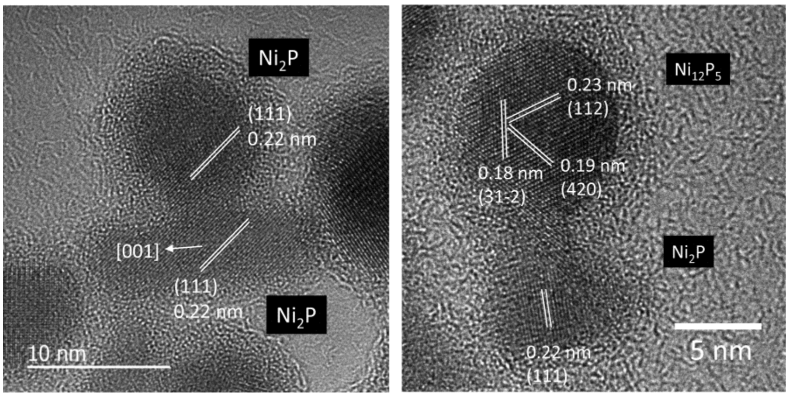

Fig. S1 Additional HRTEM images of $NiP_x@FeP_yO_z$ NPs.

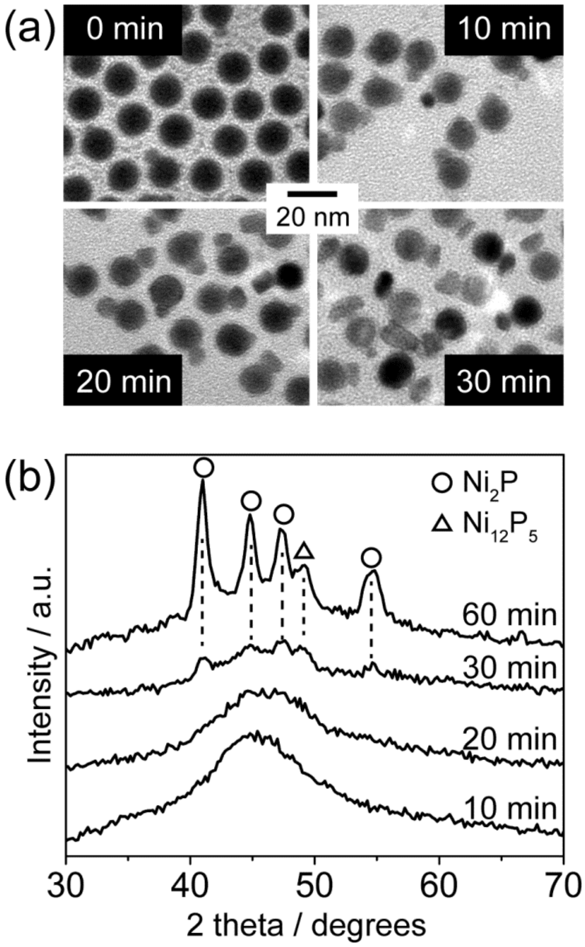

Fig. S2 (a) TEM images and (b) XRD patterns of $NiP_x@FeP_yO_z$ NPs at various reaction time.

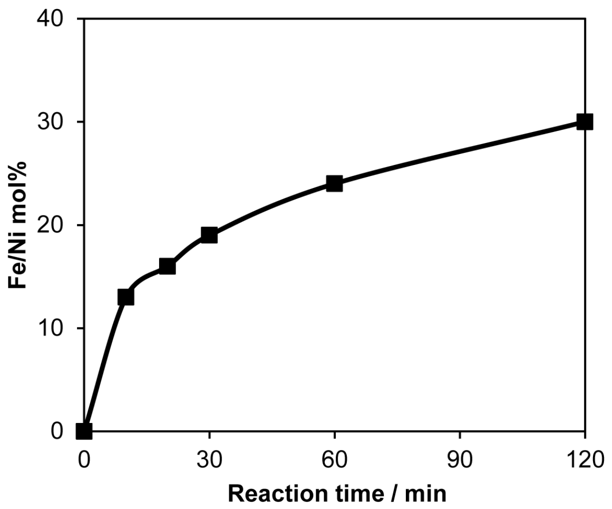

Fig. S3 Temporal change of Fe/Ni molar ratios of $NiP_x@FeP_yO_z$ NPs in the synthesis measured by XRF.

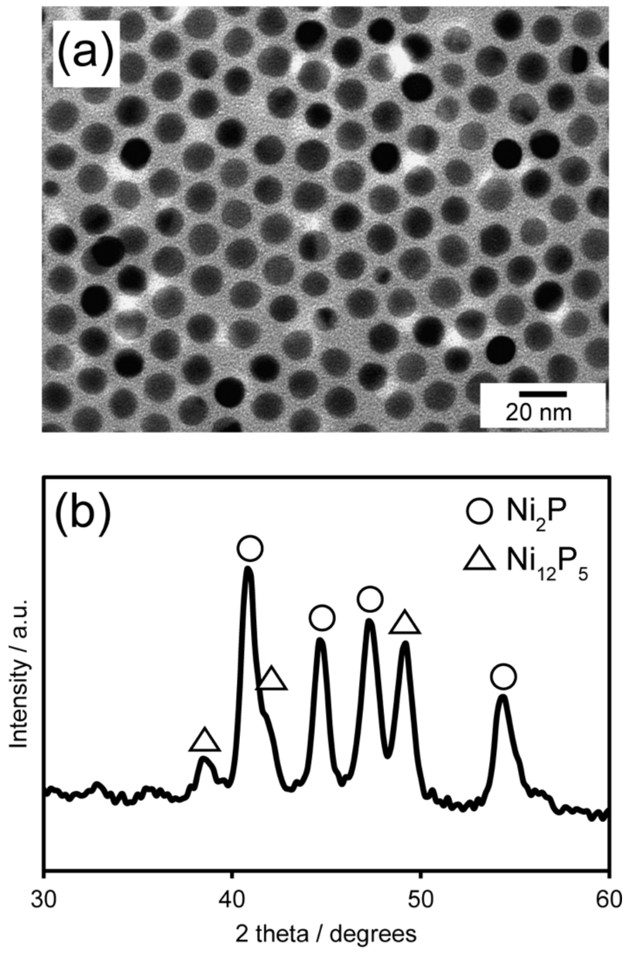

Fig. S4 (a) TEM image and (b) XRD pattern of NPs synthesized without $Fe(CO)_5$.

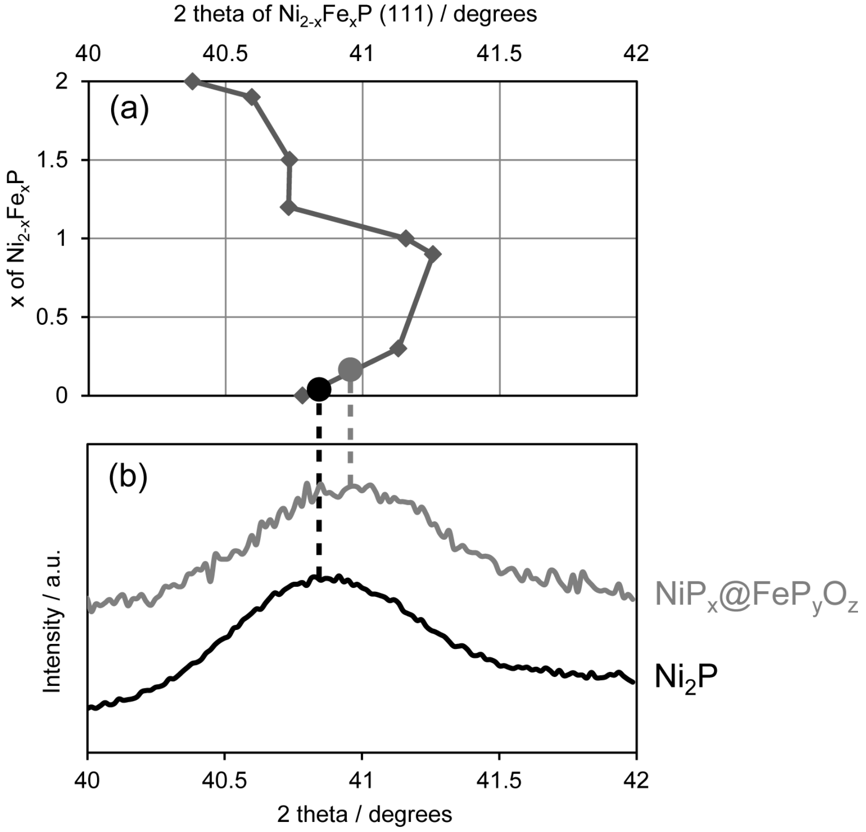

Fig. S5 (a) Change in (111) diffraction peak positions of $Ni_{2-x}Fe_xP$ phase depending on x using the data in [5]. (b) (111) diffraction patterns of Ni_2P NPs and $NiP_x@FeP_yO_z$ NPs in this work.

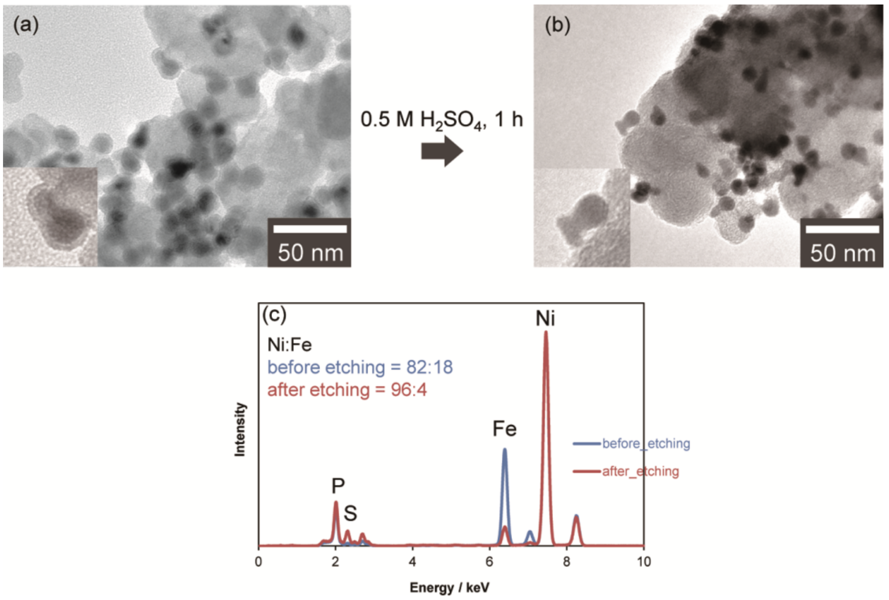

Fig. S6 TEM images of (a) NiP$_x$@FeP$_y$O$_z$ NPs and (b) shell-etched NiP$_x$ cores supported on carbon powder. (c) XRF spectra of NiP$_x$@FeP$_y$O$_z$ NPs (blue) before and (red) after etching. Inset shows the corresponding Ni:Fe molar ratios.

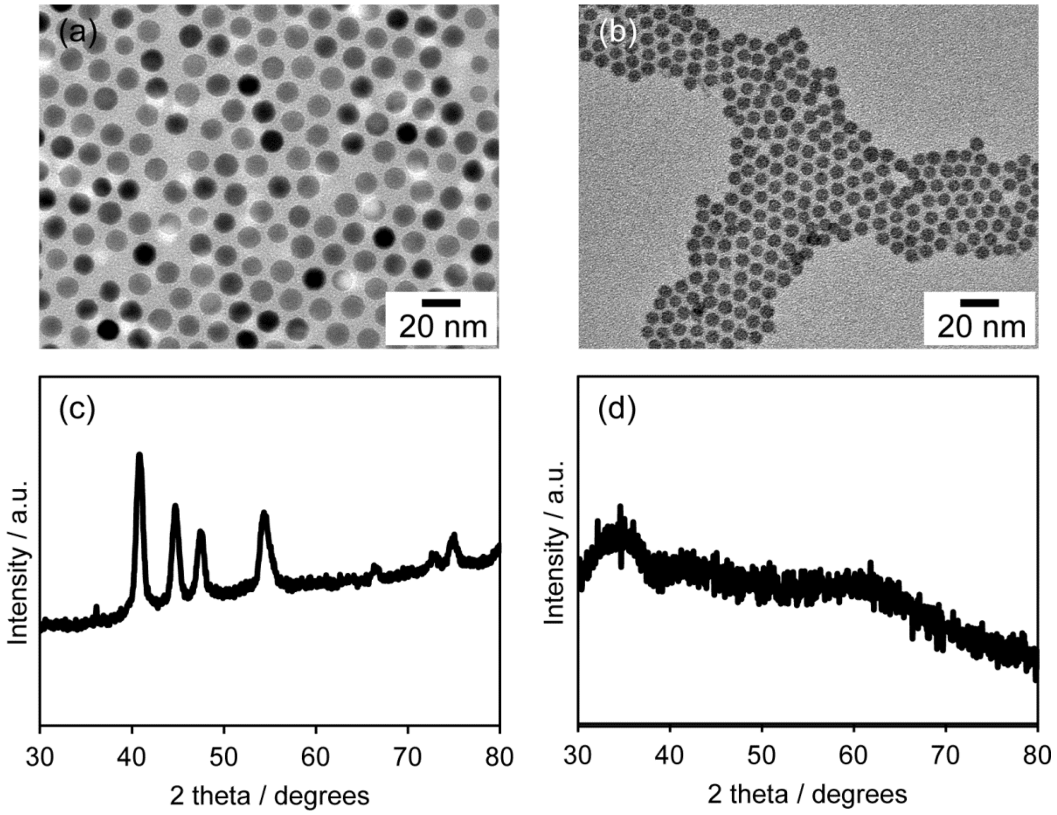

Fig. S7 (a, b) TEM images and (c, d) XRD patterns of (a, c) Ni_2P NPs and (b, d) FeO_x NPs.

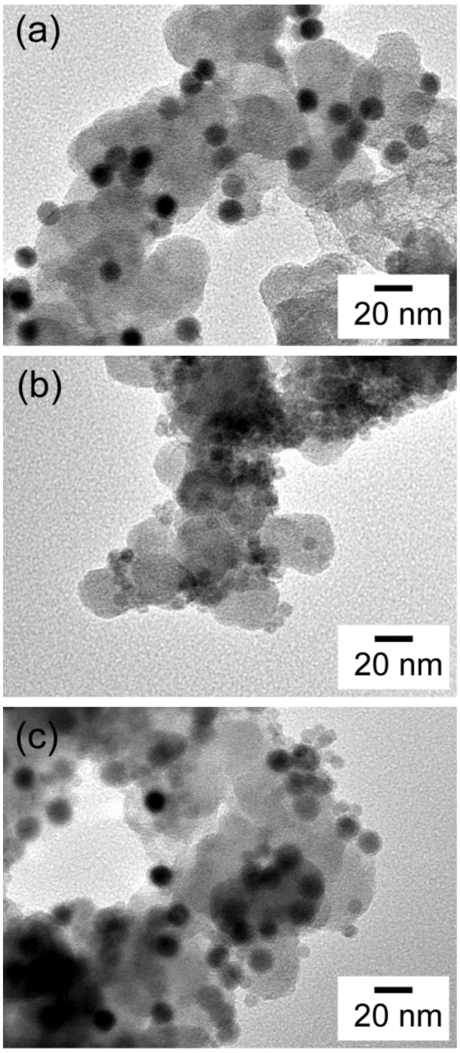

Fig. S8 TEM images of (a) Ni_2P NPs, (b) FeO_x NPs, and (c) the mixture of Ni_2P+FeO_x NPs supported on carbon powder.

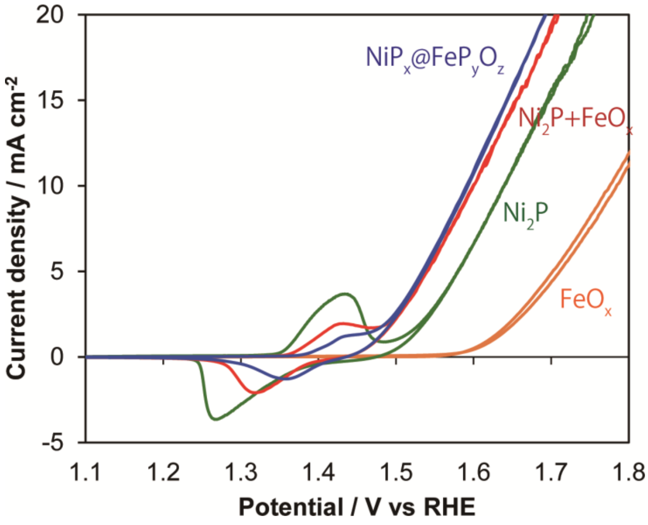

Fig. S9 Cyclic voltammograms of NiP$_x$@FeP$_y$O$_z$, FeO$_x$, Ni$_2$P, and FeO$_x$+Ni$_2$P NPs supported on carbon powder in 0.1 M KOH at 10 mV s^{-1} without *iR* compensation.

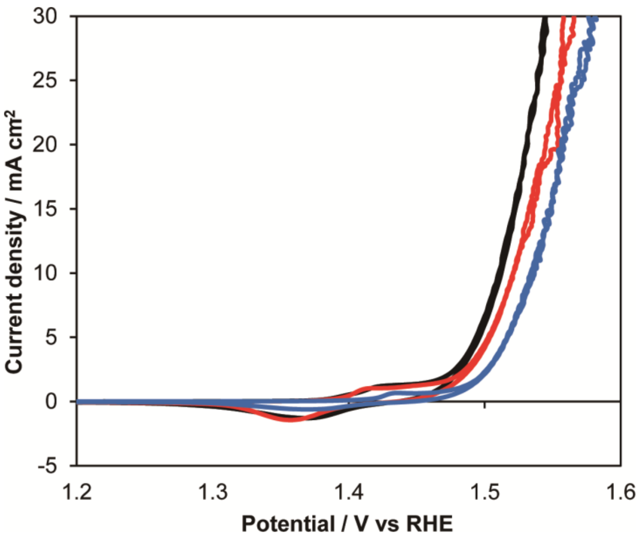

Fig. S10 Cyclic voltammograms of the NiP$_x$@FeP$_y$O$_z$ NPs at Ni/Fe molar ratio of 78/22 (black), 85/15 (red), and 72/28 (blue). The NiP$_x$@FeP$_y$O$_z$ NPs at Ni/Fe molar ratio of 85/15 78/22, and 72/28 were synthesized at 270 °C for 20, 60, and 120 min, respectively.

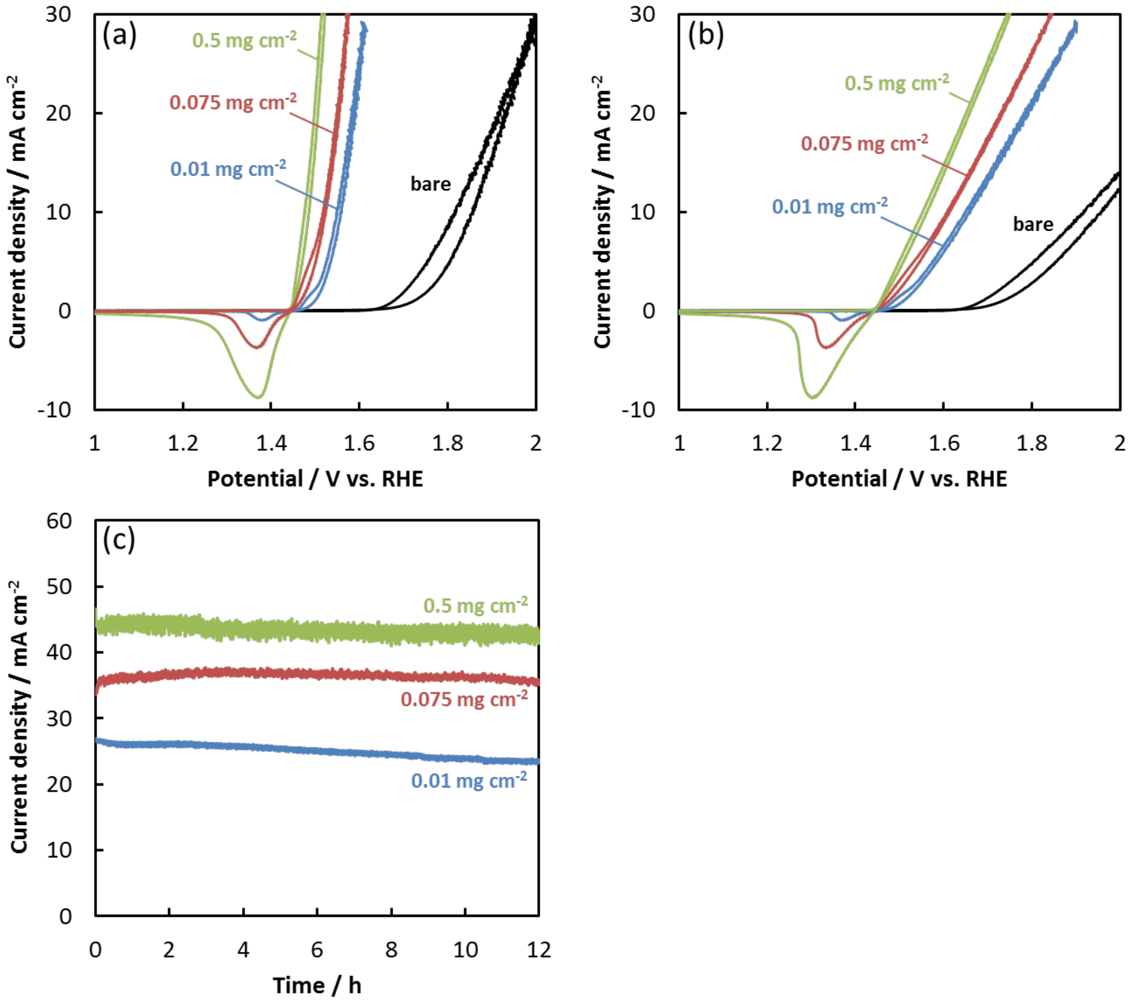

Fig. S11 Cyclic voltammograms of bare and $NiP_x@FeP_yO_z$ NPs loaded carbon papers (a) with and (b) without *iR* compensation. (c) Current density-time curves of $NiP_x@FeP_yO_z$ NPs loaded carbon paper at 1.9 V vs RHE without *iR* compensation. Electrolyte: 0.1 M KOH, RE: Hg/HgO, CE: Pt coil.

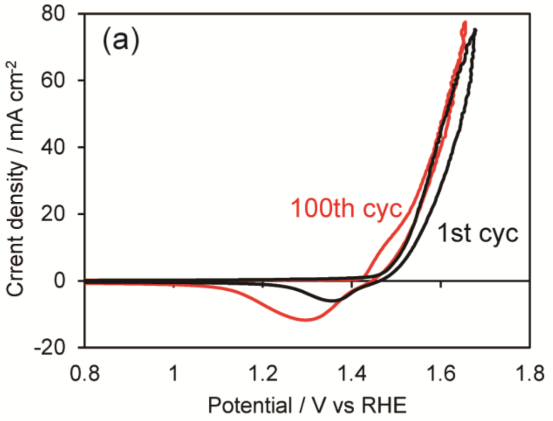

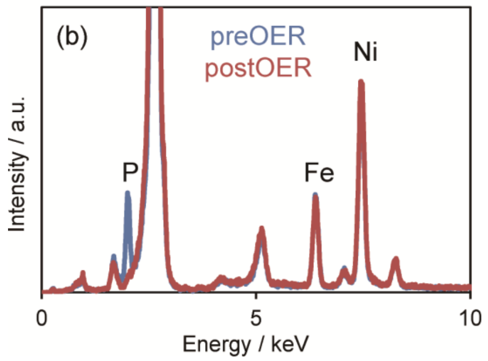

mol%	preOER	postOER
Ni	39 (81)	74 (82)
Fe	12 (19)	17 (18)
P	49	9

Fig. S12 (a) Cyclic voltammograms of the $NiP_x@FeP_yO_z$ NPs supported on carbon paper at 1st and 100th cycle in 0.1 M KOH. (b) XRF spectra of the $NiP_x@FeP_yO_z$ NPs supported on carbon paper (blue) before and (red) after 100 cycles CV in 0.1 M KOH. A table shows the molar ratios of Ni:Fe:P and the molar ratios of Ni:Fe in parentheses.

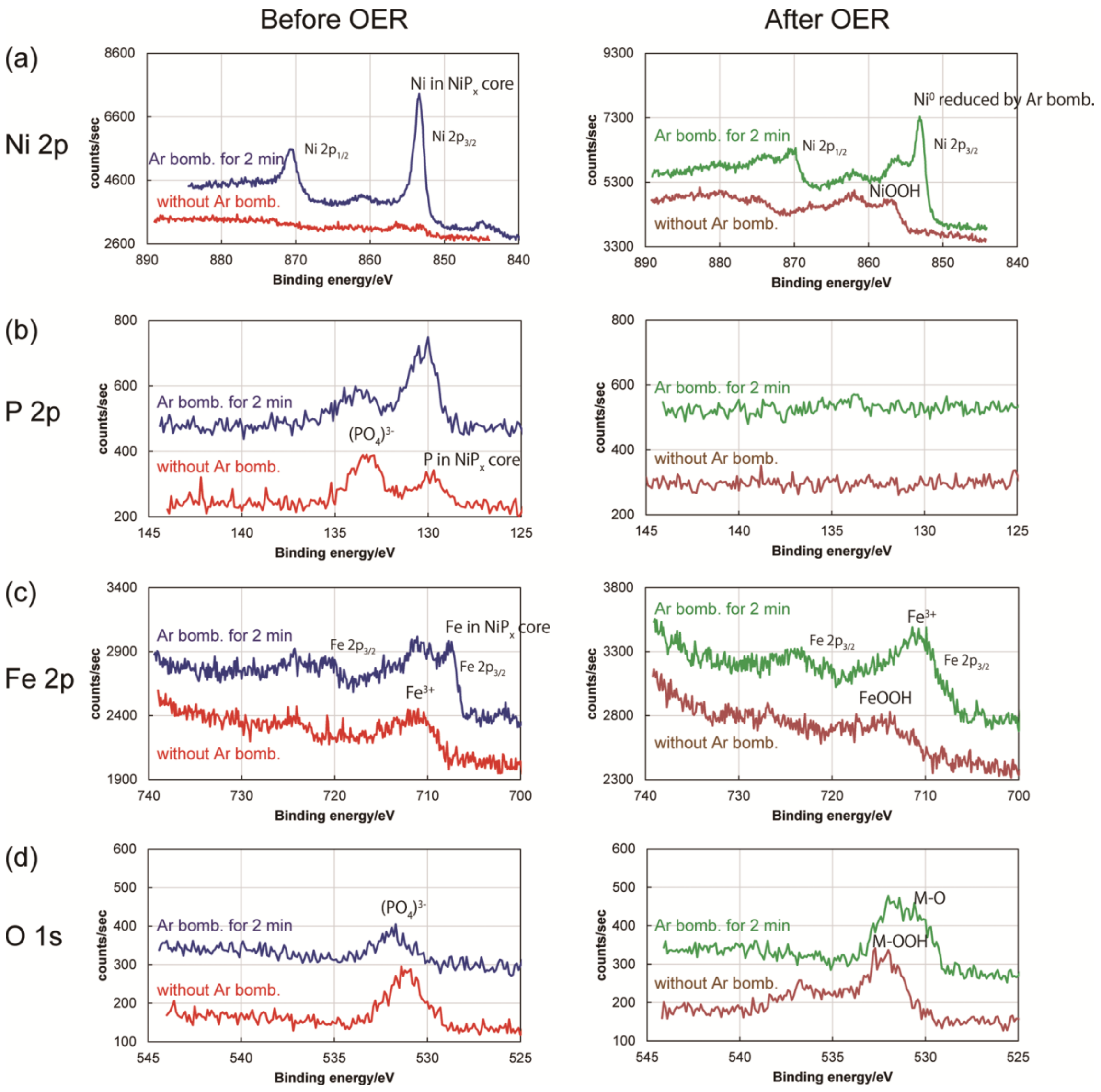

Fig. S13 XPS spectra of the $NiP_x@FeP_yO_z$ NPs supported on carbon paper before and after 100 cycles OER in 0.1 M KOH. (a) Ni 2p, (b) Fe 2p, (c) P 2p, (d) O 1s. Each binding energy was calibrated by C 1s peak (284.6 eV). The Ar bombardment was carried out at 500 eV of the beam energy for 2 min.

Effect of Ar bombardments

(a) Ni 2p: *Before OER*; after Ar bombardment, strong peaks of Ni^0, which corresponded to Ni in the NiP_x core appeared. *After OER*; the Ar bombarded sample also showed Ni^0 peaks, which were attributed to reduced Ni cations in NiOOH formed during the Ar bombardment.

(b) P 2p: *Before OER*; after Ar bombardment, P^0 peak at 130 eV became strong owing to the exposure of P^0 in NiP_x cores. *After OER*; no peaks appeared even after Ar bombardment, indicating P was not only absent from the surface but was also eliminated from the bulk during OER.

(c) Fe 2p: *Before OER*; after Ar bombardment, a weak Fe^0 peak emerged at 707.5 eV, which was attributed to Fe(0) in NiP_x cores. *After OER*; no peaks of Fe^0 appeared because most of the NiP_x cores were oxidized during OER.

(d) O 1s: *Before OER*; after Ar bombardment, O1s peak slightly shifted. One explanation for this shift is that the outer and inner parts of the shell were mainly oxide (531 eV) and phosphate (532 eV), respectively. After OER, after Ar bombardment, a shoulder peak at a lower binding energy (530 eV) assigned to metal oxide appeared, indicating that the inner part of the sample after OER contained metal oxide and (Ni, Fe)O_xH.

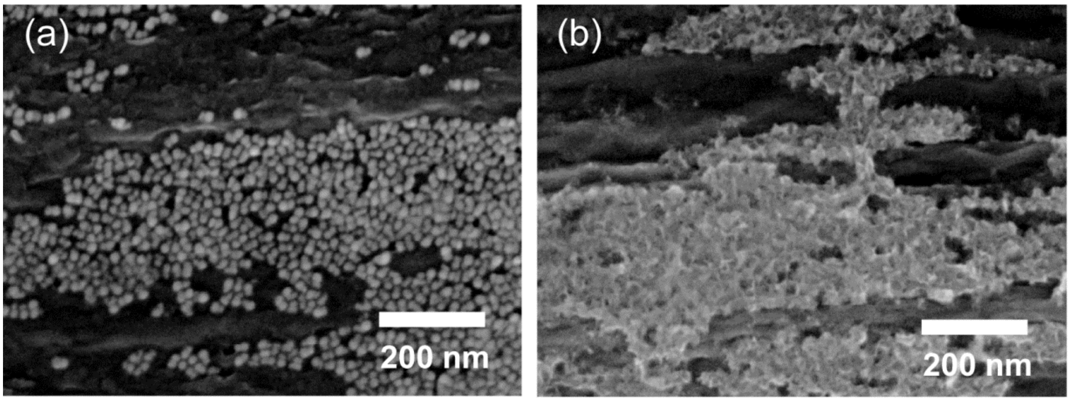

Fig. S14 SEM images of the $NiP_x@FeP_yO_z$ NPs supported on carbon paper (a) before and (b) after 100 cycles CV in 0.1 M KOH.

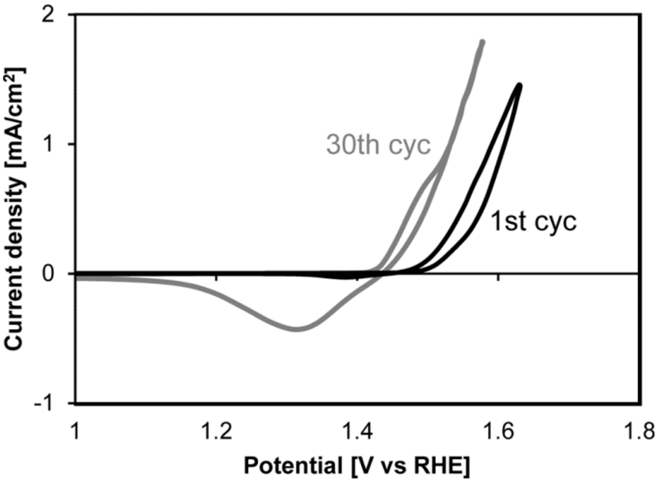

Fig. S15 (a) Cyclic voltammograms of the $NiP_x@FeP_yO_z$ NPs loaded on FTO coated glass at 1st and 30th cycle in 0.1 M KOH.

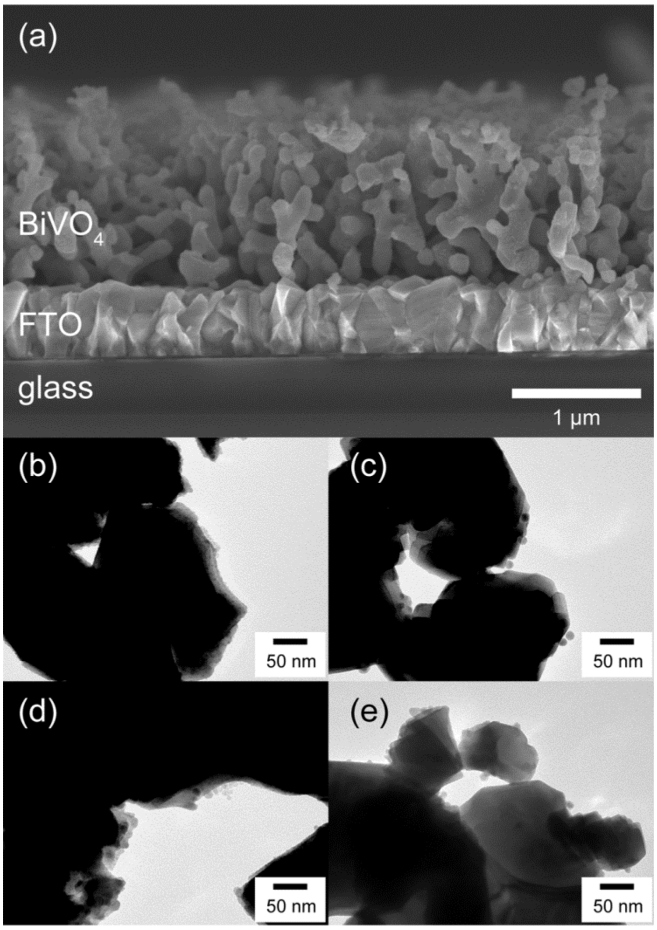

Fig. S16 (a) Cross sectional SEM image of the $BiVO_4$ photoelectrode. TEM images of (b) FeO_x NPs, (c) Ni_2P NPs, (d) the mixture of Ni_2P+FeO_x NPs, and (e) NiP_x@FeP_yO_z NPs loaded on $BiVO_4$ photoelectrodes.

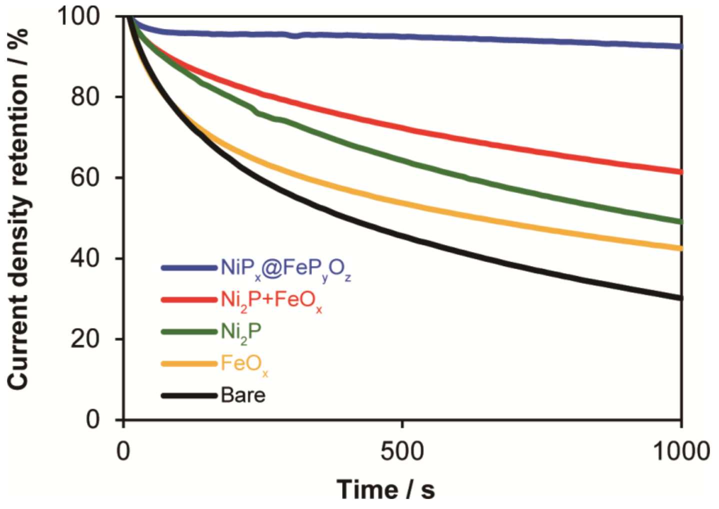

Fig. S17 Current density durability of bare and NPs loaded $BiVO_4$ in CA measurements @1.23 V vs RHE.

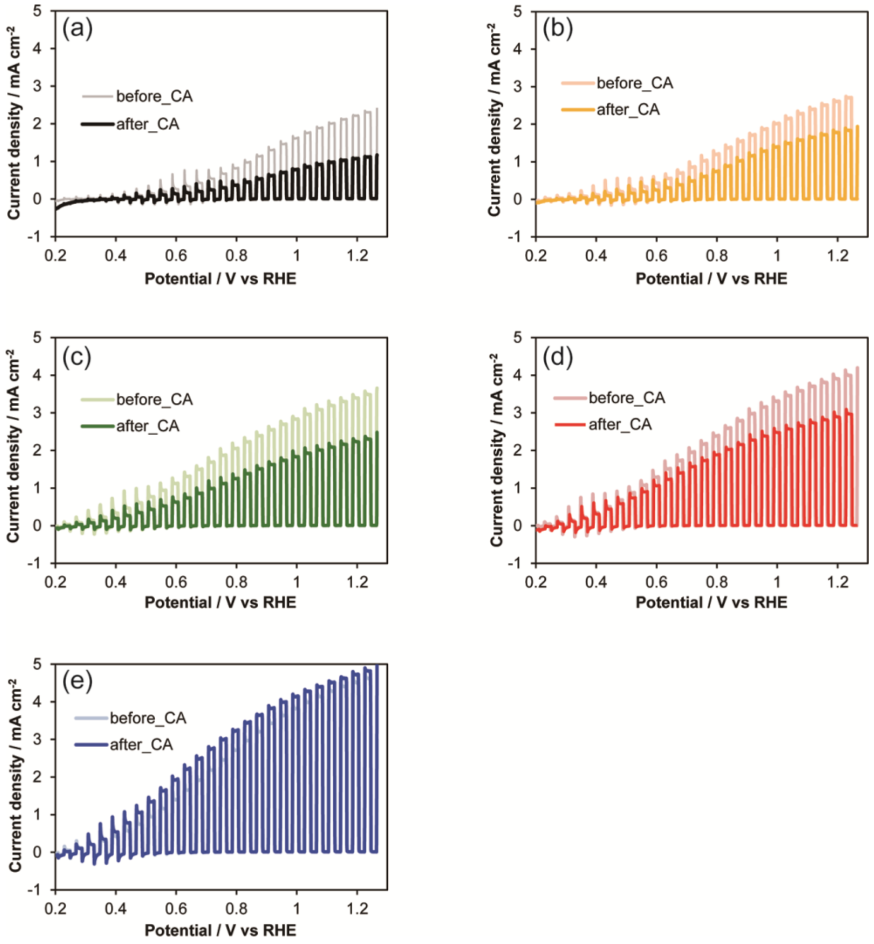

Fig. S18 Photocurrent density curves of (a) bare $BiVO_4$ and (b) FeO_x, (c) Ni_2P, (d) Ni_2P+FeO_x and (e) $NiP_x@FeP_yO_z$ NPs loaded $BiVO_4$ before and after CA measurements.

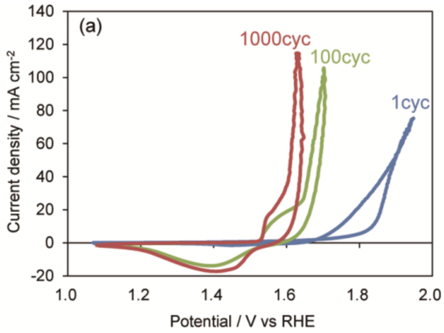

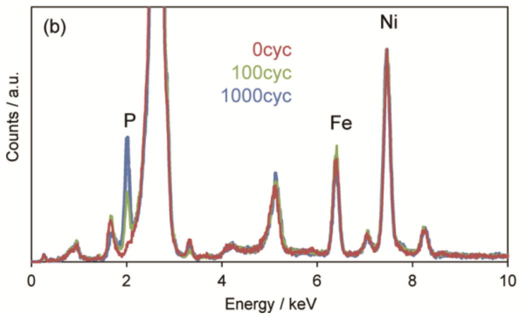

mol%	0cyc	100cyc	1000cyc
Ni	38 (81)	48 (79)	73 (79)
Fe	12 (19)	16 (21)	19 (21)
P	50	36	8

Fig. S19 (a) Cyclic voltammograms and (b) XRF spectra of the NiP_x@FeP_yO_z NPs supported on carbon papers at 0, 100, and 1000 cycles in 0.125 M $K_2B_4O_7$ at 100 mV s^{-1}. A table shows the molar ratios of Ni:Fe:P and the molar ratios of Ni:Fe in parentheses. Decreased P molar ratio after CV suggests that the NiP_x@FeP_yO_z NPs transformed into the (Ni, Fe)O_xH_y during OER even in 0.125 M $K_2B_4O_7$.

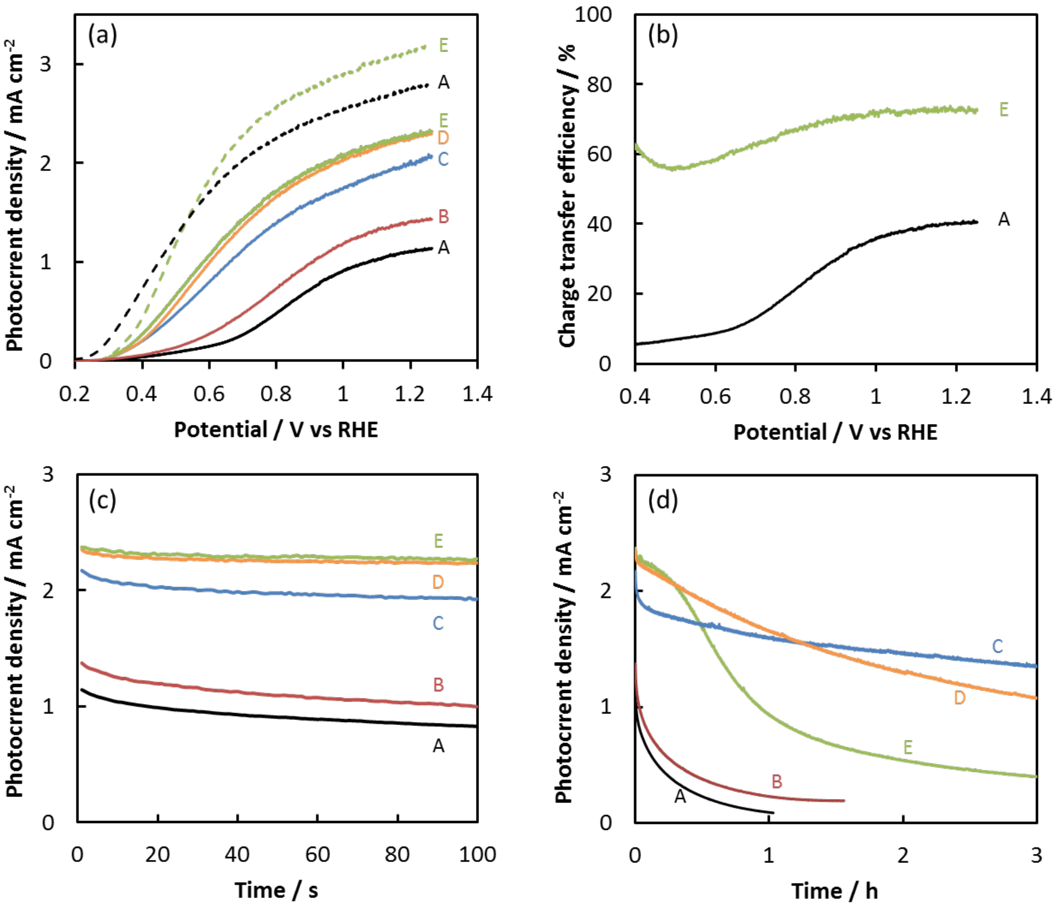

Fig. S20 (a) LSV of bare and various amounts of NiP$_x$@FeP$_y$O$_z$ NPs loaded $BiVO_4$ under simulated sunlight (100 mW cm^{-2}). (b) Charge transfer efficiency of samples A and E. Current-time curves of $BiVO_4$ electrodes after (c) 100 s and (d) 3 h (10800 s) at 1.23 V vs RHE.

(A) Bare and (B) 0.05, (C) 0.25, (D) 1, and (E) 5 mg mL^{-1} NPs ink spin-casted $BiVO_4$.

Solid lines: in 0.125 M $K_2B_4O_7$, Dashed lines: in 0.5 M potassium phosphate buffer with 1 M Na_2SO_3, RE: Ag/AgCl, CE: Pt coil.

Table S1 Comparison of OER activities of various transition metal phosphide based OER electrocatalysts

Catalyst	Morphology	Electrolyte	Overpotential @10 mA cm^{-2} / V	loading amount / mg cm^{-2}	Ref.
NiP_x@FeP_yO_z on carbon powder	~20 nm NP	0.1 M KOH	0.28	0.02	This work
NiP_x@FeP_yO_z on carbon paper			0.32	0.01	This work
			0.29	0.075	This work
			0.25	0.5	This work
CoFeP	urchin shape 80 x 5 nm	0.1 M KOH<br>1 M KOH	0.37<br>0.28	0.10	[6]
CoP/C	20 nm rods	0.1 M KOH	0.36	0.05	[7]
CoMnP	~5 nm NP	1 M KOH	0.33	0.284	[8]
CoP	1-5 µm needle film	1 M KOH	0.27	4.95	[9]
NiFeP	a few µm thick film	1 M KOH	0.28	a few mm thickness	[10]
Ni phosphides	>300 nm nanoporous solid	1 M KOH	0.32	0.15	[11]
Ni_5P_4	40 µm thick film	1 M KOH	0.29	11.71	[12]
Ni_2P	50 nm solid	1 M KOH	0.29	0.14	[13]
Fe doped Ni_2P	1 µm nanosheet	1 M KOH	<0.2	1.4	[14]
NiFe-OH/NiFeP	several µm nanosheet	1 M KOH	0.199	1.8	[15]
Fe_3O_4@NiP_x	~10 nm NP	1 M KOH	0.26	0.2	[16]
GDs/$Co_{0.8}Ni_{0.2}P$	~200 nm porous tubular	1 M KOH	0.287	no data	[17]
Mg/Ni_2P	thin film	1 M KOH	0.28	1.33	[18]
FeP-FeP_xO_y	100 x 800 nm hollow	1 M KOH	0.28	0.3	[19]
$Co_{0.63}Fe_{0.21}P_{0.16}$	200-500 nm sphere	1 M KOH	0.21	0.2	[20]
NiFe(3:1)-P	flower-like 1-1.5 µm	1 M KOH	0.233	0.1	[21]

Table S2 Comparison of performance of cocatalyst-modified nondoped-$BiVO_4$ photoelectrode under simulated sunlight.

Cocatalyst	Deposition method	Photocurrent @1.23V /mA cm^{-2}	Photocurrent retension	Ref.
CoPi	PED[a]	6.1	90% at $0.6V_{RHE}$ in 6h	[22]
Co_4O_4 Cubane	dropcasting	5	~50% at $0.7V_{RHE}$ in 4h	[23]
β-FeOOH	impregnation	4.3	100% at $1V_{RHE}$ in 2h	[24]
FeOOH	PED[a]	4.2	100% at 0.6V vs CE in 48h	[25]
CoOOH	nanosheet dropcasting	4	97% at $1V_{RHE}$ in 4h	[26]
NiB	NPs dropcasting	3.47	~50% at $1.23V_{RHE}$ in 1.1h	[27]
In_2O_3/CoOOH	CBD[b]+PEC deposition	3.4	~85% at 1.23 V_{RHE} in 1h	[28]
$ZnFe_2O_4/Co^{2+}$	PED[a]+immersion	2.84	~75% at $1.23V_{RHE}$ in 0.83h	[29]
$ZnFe_2O_4$	PED[a]	2.76	~60% at $1.23V_{RHE}$ in 0.83h	[29]
Co^{2+}	immersion	1.53	~10% at $1.23V_{RHE}$ in 0.83h	[29]
Co_3O_4	NPs dropcasting+annealing	2.71	~80% at $0.6V_{RHE}$ in 0.1h	[30]
Ni:CoO_x	electrospray pyrolysis	2.62	~40% at 0.77 V_{RHE} in 2h	[31]
$NiP_x@FeP_yO_z$	NPs dropcasting	2.32	17% at $1.23V_{RHE}$ in 3h	this work
$NiP_x@FeP_yO_z$	NPs dropcasting	2.28	46% at $1.23V_{RHE}$ in 3h	this work
$NiP_x@FeP_yO_z$	NPs dropcasting	2.02	62% at $1.23V_{RHE}$ in 3h	this work
$CoLa(OH)_x$	electrodeposition	2	93% at 0.8V vs CE in 1.1h	[32]
Ru-complex	dip-coating	1.4	~40% at $1.23V_{RHE}$ in 1.8h	[33]
CoPi	electrodeposition	1.4	~80% at $1.23V_{RHE}$ in 1.8h	[33]
CoFe-PB	immersion	1.1	~45% at $1.23V_{RHE}$ in 15 h	[34]

[a]PED = Photo-assisted electrodeposition

[b]CBD = Chemical bath deposition

Gray rows: $BiVO_4$ is fully covered with robust cocatalyst layer

White rows: $BiVO_4$ is partially covered with nanosized particle or monolayer of molecule

References

[1] S. Akiyama *et al.*, *Small*, 2016, **12**, 5468.

[2] H. Kura *et al.*, *J. Phys. Chem. C*, 2010, **114**, 5835.

[3] T. W. Kim *et al.*, *Science*, 2014, **343**, 990.

[4] P. He *et al.*, *Anal. Chem.*, 1986, **58**, 517.

[5] A. H. Mudiyanselage *et al.*, *Chem. Mater.*, 2015, **27**, 6592.

[6] A. M. Garcia *et al.*, *Angew. Chem. Int. Ed.*, 2015, **54**, 9642.

[7] J. Ryu *et al.*, *ACS Catal.*, 2015, **5**, 4066.

[8] D. Li *et al.*, *J. Am. Chem. Soc.*, 2016, **138**, 4006.

[9] P. Wang *et al.*, *ChemSusChem*, 2016, **9**, 472.

[10] C. G. Read *et al.*, *ACS Appl. Mater. Interfaces*, 2016, **8**, 12798.

[11] J. Li *et al.*, *ACS Appl. Mater. Interfaces*, 2016, **8**, 10826.

[12] M. Ledendecker *et al.*, *Angew. Chem. Int. Ed.*, 2015, **54**, 12361.

[13] L. A. Stern *et al.*, *Energy Environ. Sci.*, 2015, **8**, 2347.

[14] J. Wang *et al.*, *Inorg. Chem.*, 2017, **56**, 1041.

[15] H. Liang *et al.*, *ACS Energy Lett.*, 2017, **2**, 1035.

[16] A. Dutta *et al.*, *ACS Energy Lett.*, 2018, **3**, 141.

[17] J. Hou *et al.*, *ACS Appl. Mater.*, *Interfaces* 2017, **9**, 24600.

[18] J. Xu *et al.*, *ACS Catal.*, 2017, **7**, 5450.

[19] J. Xu *et al.*, *ACS Appl. Nano Mater.*, 2018, **1**, 617.

[20] T. Wang *et al.*, *J. Mater. Chem. A*, 2017, **5**, 25378.

[21] P. Li *et al.*, *J. Mater. Chem. A*, 2018, **6**, 2231.

[22] H. S. Han *et al.*, *Energy Environ. Sci.*, 2018, in press.

[23] Y.Wang *et al.*, *Angew. Chem. Int. Ed.*, 2017, **56**, 6911.

[24] B. Zhang *et al.*, *Angew. Chem. Int. Ed.*, 2018, **57**, 2248.

[25] T. W. Kim *et al.*, *Science*, 2014, **343**, 990.

[26] F. Tang *et al.*, *ACS Appl. Mater. Interfaces*, 2018, **10**, 6228.

[27] K. Dang *et al.*, *Nanoscale*, 2017, **9**, 16133.

[28] W. Qiu *et al.*, *J. Phys. Chem. C*, 2017, **121**, 17150.

[29] T. W. Kim *et al.*, *J. Phys. Chem. Lett.*, 2016, **7**, 447.

[30] X. Chang *et al.*, *J. Am. Chem. Soc.*, 2015, **137**, 8356.

[31] Y. Liu *et al.*, *J. Phys. Chem. C*, 2016, **120**, 23449.

[32] M. Chhetri *et al.*, *ACS Energy Lett.*, 2017, **2**, 1062.

[33] M. de Repinis *et al.*, *J. Phys. Chem. C*, 2015, **119**, 7275.

[34] F. S. Hegner *et al.*, *ACS Appl. Mater. Interfaces*, 2017, **9**, 37671.
